# The impact of obesity or overweight on the risk of glaucoma: a meta-analysis

**DOI:** 10.3389/fmed.2026.1756819

**Published:** 2026-01-30

**Authors:** Piao Xie, Hanze Qiu, Luyu Zhao, Dahai Hu, Hui Tang, Rongzhao Lu, Hongwei Pan

**Affiliations:** 1 Department of Ophthalmology, The First Affiliated Hospital of Jinan University, Jinan University, Guangzhou, China; 2 Program in Data Science, New York University Shanghai, Shanghai, China; 3 Department of Ultrasound, The First Affiliated Hospital of Jinan University, Jinan University, Guangzhou, China; 4 Guangdong Provincial Key Laboratory of Speed Capability, The Guangzhou Key Laboratory of Precision Orthopedics and Regenerative Medicine, Department of Sports Medicine, The First Affiliated Hospital of Jinan University, Jinan University, Guangzhou, China; 5 The Clinical Medicine Research Institute, The First Affiliated Hospital of Jinan University, Jinan University, Guangzhou, China; 6 The Clinical Medicine Research Institute, The Fifth Affiliated Hospital of Jinan University, Jinan University, Heyuan, China; 7 Department of Breast Surgery, The First Affiliated Hospital of Jinan University, Jinan University, Guangzhou, China

**Keywords:** glaucoma, intraocular pressure, meta-analysis, obesity, overweight

## Abstract

**Background:**

Glaucoma is a major cause of vision loss. Preventing the development of glaucoma has become a focus of attention. Whether obesity or overweight is related to the glaucoma development still remains a matter of debate.

**Objective:**

The purpose of this study is to explore the impact of obesity or overweight on the risk of glaucoma.

**Materials and methods:**

A systematic search was conducted in the PubMed, Embase, and Web of Science databases for relevant studies published up to 24 March, 2025. Original studies reporting the relationship between obesity or overweight and glaucoma risk were selected. Clinical outcomes were evaluated based on odds ratios (OR) or hazard ratios (HR) with corresponding 95% confidence intervals (CI). The study quality was assessed using an eight-component rating scale.

**Results:**

Thirteen studies were included. Individuals with obesity or overweight had a 60% higher risk of glaucoma compared with the normal-weight population (OR: 1.60 [95% CI: 1.19–2.17], *p* = 0.002), while the association was not significant for HR values (HR: 1.14 [95% CI: 1.00–1.31], *p* = 0.058). Subgroup analysis revealed that individuals with obesity or overweight in developed countries had a 91% higher risk of glaucoma (OR: 1.91 [95% CI: 1.44–2.52], *p* < 0.001). In addition, obesity or overweight in men was associated with a higher risk of glaucoma (OR: 2.14 [95% CI: 1.24–3.69], *p* = 0.006).

**Conclusion:**

Obesity or overweight may increase the risk of glaucoma in term of OR values. Obese or overweight individuals in developed countries, especially obese men, appear to be associated with a higher risk of glaucoma. Maintaining weight within the normal range may be an effective measure to prevent glaucoma.

## Introduction

Glaucoma is a disease that leads to irreversible vision loss and its main characteristic is the progressive degeneration of retinal ganglion cells, often accompanied by increased intraocular pressure (1–3). Glaucoma is primarily classified into open-angle glaucoma and angle-closure glaucoma, with approximately half of the glaucoma-related blindness attributable to angle-closure glaucoma (4). Other subtypes include congenital glaucoma and secondary glaucoma. Because glaucoma progresses slowly and neural mechanisms compensate for the areas of vision loss, patients often remain unaware of vision impairment until the late stages of the disease (5–7). At present, no effective treatment exists to restore vision loss caused by glaucoma. Therefore, effective case detection and preventive strategies are essential.

The prevalence of obesity has increased significantly over the past 30 years, mainly due to people’s dietary habits (8–10). Obesity or overweight negatively impact health. Obesity is well known to negatively impact both the cardiovascular and metabolic systems (11, 12). Meanwhile, obesity is also one of the main risk factors for diseases such as coronary heart disease, type 2 diabetes, hypertension, stroke, dyslipidemia, osteoarthritis, and sleep apnea (13, 14).

Currently, there are relatively few studies on the impact of obesity or overweight on the development of eye diseases. Whether obesity or overweight substantially increases the risk of glaucoma remains a focus of attention. A clinical study reported a harmful link between obesity and vision, although the specific mechanisms and consequences of this association are still unclear (15). Other studies have shown that obesity may be related to glaucoma development (16, 17). Liu et al. showed in a meta-analysis that obesity increases the risk of elevated intraocular pressure, but does not significantly affect the occurrence of open-angle glaucoma (18). In contrast, Jung et al. showed that obesity and metabolic health status are closely associated with an increased risk of open-angle glaucoma (16). Therefore, whether obesity or overweight is related to glaucoma risk remains debated, and further research is necessary.

This study conducted a meta-analysis on the relationship between obesity or overweight and the risk of glaucoma. The study also performed subgroup analyses for different countries, ages, and sexes to investigate the impact of various factors on the risk of glaucoma.

## Materials and methods

### Search strategy

This study was conducted in accordance with the preferred reporting items for systematic reviews and meta-analyses (PRISMA) (19). Two authors (PX and DHH) systematically and independently searched for relevant literature using the PubMed, Web of Science, and Embase databases up to 24 March, 2025. The search strategy included the following terms: “obesity” or “nutritional and metabolic diseases” or “nutrition disorders” or “overnutrition” OR “overweight” OR “obesity hypoventilation syndrome” OR “pediatric obesity” OR “pregnancy in obesity” OR “intra-abdominal fat” OR “body mass index” OR “BMI” AND “glaucoma” OR “intraocular pressure” OR “ocular hypertension” OR “open-angle glaucoma” OR “normal tension glaucoma” OR “high tension glaucoma.” In addition, the reference lists of the retrieved meta-analyses may be screened for additional eligible articles. No other restrictions were imposed.

### Screening criteria

The inclusion criteria were as follows: (1) literature reported the relationship between obesity or overweight and the risk of glaucoma or elevated IOP; (2) cohort, case–control, or cross-sectional study design was adopted; (3) relevant risk outcomes, such as hazard ratio (HR), odds ratio (OR), or relative risk (RR), were further analyzed in this study; and (4) study was published in English.

The exclusion criteria were as follows: (1) conference abstracts, letters, or editorials; (2) published in a language other than English; (3) study subjects were non-human; and (4) studies involving the effects of relevant drug treatments or special situations (such as ophthalmic surgery).

### Data extraction

The two authors (PX and DHH) independently extracted the data, and any discrepancies were resolved by consulting a third researcher. The data extracted from each included study comprised the first author, year, country, number of participants, proportion of women, age, study design, participant status, obesity assessment, and relevant outcomes (HR, OR, or RR, along with 95% confidence intervals (CI)). Obesity, overweight, and glaucoma are defined as shown in Table 1.

**Table 1:** Definitions of obesity, overweight, and glaucoma included in this study.

Author	Obesity or overweight definition	Glaucoma definition
Fujita (23)	Overweight or obese (BMI ≥ 25 kg/m^2^)	ICD-10 codes, H40.1-H40.9, H42.0, H42.8, Q15.0
Ngo (24)	Overweight: BMI (25.0–29.9), Obese: BMI ≥ 30.	NR
Chen (25)	Obesity or morbid obesity (ICD-9-CM codes 278.0, 278.00, 278.01)	ICD-9-CM codes 365.1, 365.10, 365.11, 365.12
Rosa (26)	BMI: low (<18.5), moderate (18.5–24.9), high (25.0–29.9), and very high (≥30).	NR
Lee (27)	BMI ≥ 25 kg/m^2^	NR
Lin (28)	Lower BMI (<19 kg/m^2^) Normal BMI (19–24.9 kg/m^2^)	NR
Zhao (29)	Obesity was defined as a BMI of 24.0 kg/m^2^ or higher.	Self-reports
Wise (30)	BMI ≥ 35 kg/m^2^	Self-reports
Pasquale (31)	BMI ≥ 30 kg/m^2^	Self-reports
Ko (32)	BMI ≥ 30 kg/m^2^	Cup-to-disc ratio (CDR)
Cohen (33)	BMI ≥ 25 kg/m^2^	NR
Jang (34)	BMI ≥ 25 kg/m^2^	NR
Kyari (35)	Overweight or obese (BMI ≥ 25 kg/m^2^)	International Society for Geographical and Epidemiological Ophthalmology (ISGEO) criteria

NR, no report; BMI, body mass index.

### Evaluation of study quality

This study used a modified eight-point assessment scale to evaluate the quality of the included studies (20). The scale consists of six parts: selection bias, study design (such as cross-sectional or cohort), confounding factors (such as age or sex control), data collection methods, and data extraction and analysis. Each component was graded as weak, moderate, or strong. The overall study quality was divided into weak quality (≥2 components rated as weak), moderate quality (<3 components rated as strong, and <2 components rated as weak), and strong quality (≥3 components rated as strong and ≤1 components rated as weak).

### Statistical analysis

The HR, OR, and their corresponding 95% CI were used to assess the relationship between obesity or overweight and glaucoma. A random-effects model was used, and Stata version 15 (StataCorp LLC, College Station, TX, United States) was employed to conduct the meta-analysis of effect sizes (HR/OR) and their corresponding 95% CI.

Furthermore, the prediction intervals (21) for the relevant ORs was calculated using the following formula:


$$\text{Prediction intervals}=\ln(\mathrm{HR}/\text{OR})\pm (Z_{0.975}\times \mathrm{SE}\times \sqrt{2})$$

where SE = {ln(upper) − ln(lower)}/(2 × Z_0.975_) and Z_0.975_ ≈ 1.96.

In addition, stratified subgroup analyses were conducted based on sex, country (developed countries or developing countries), and age (≥40 years). The I^2^ statistic was used to assess the heterogeneity of the results. The Egger’s and Begg’s tests were employed to evaluate publication bias (22). Finally, apart from heterogeneity, a *p*-value of less than 0.05 was considered statistically significant.

## Results

**Figure 1:**
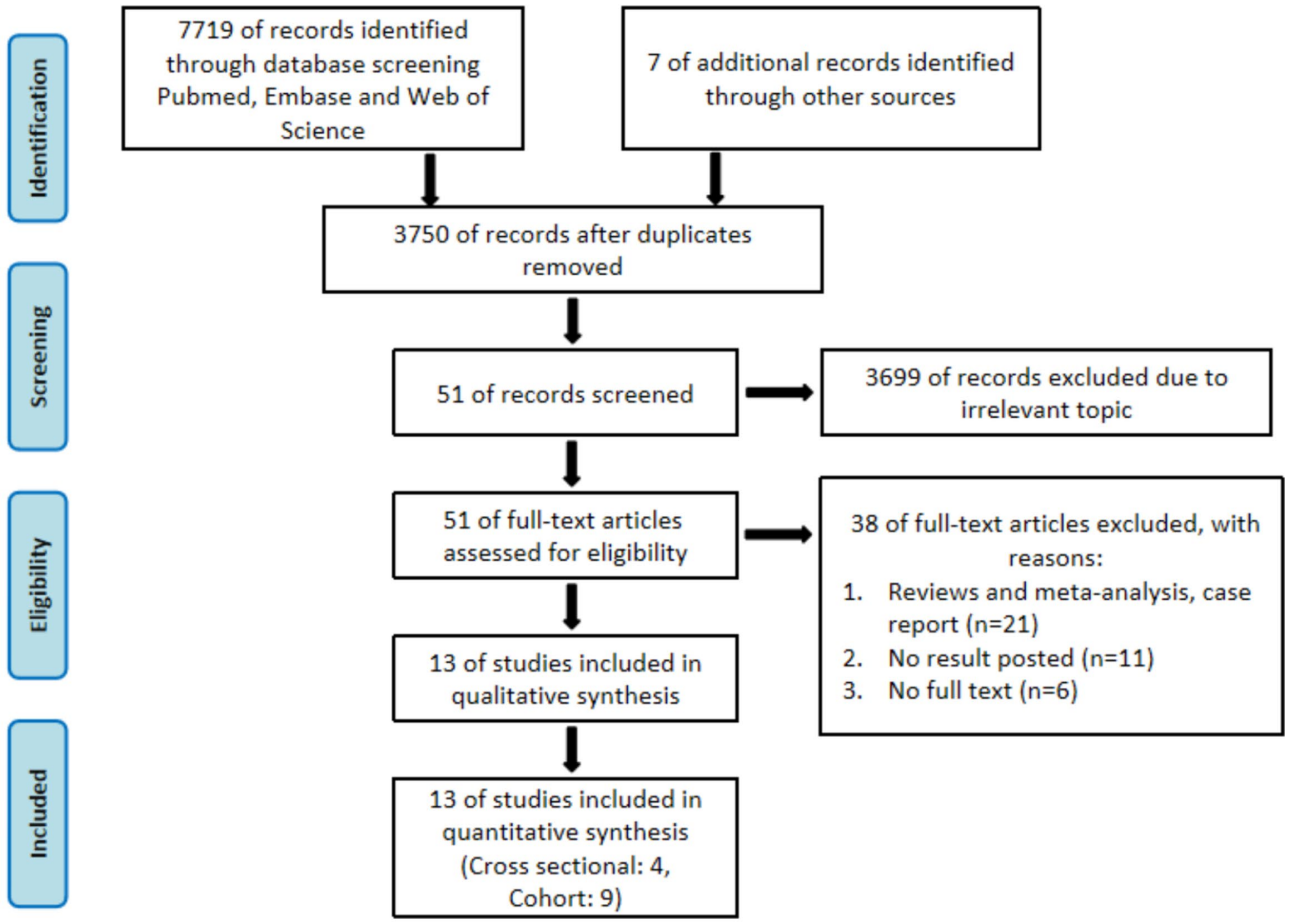
The flow chart of screening studies.

Initially, 7,719 relevant studies were identified, and seven additional studies were supplemented from reference lists. After excluding 3,976 duplicate studies, 3,699 irrelevant studies were excluded through abstract and title screening, leaving 51 studies for full-text assessment. Based on the inclusion and exclusion criteria, 38 studies were excluded, including reviews, meta-analyses, or case reports (*n* = 21); studies without relevant outcomes (*n* = 11); and studies without available full text (*n* = 6). Finally, a total of 13 studies were included in the meta-analysis (23–35) (Figure 1).

### Study characteristics

Among the 13 studies included in this research, 4 were cross-sectional, 4 were prospective cohort, and 5 were retrospective cohort studies. After quality assessment of the literature, 84.6% of the studies were rated as medium quality, 7.7% as high quality, and 7.7% as weak quality. In terms of sample size, 12 studies exceeded 5,000 participants, while only one study had less than 5,000 participants (Table 2).

**Table 2:** Characteristics of the included studies.

Author	Year	Country	Number of patients	Percentage of women (%)	Age	Study design	Patient’s condition	Outcomes	Quality assessment
Fujita (23)	2023	Japan	3,110,743	38.3	44.4	Retrospective	Excluding individuals aged <20 years, those with missing data and those with pre-existing glaucoma, defined as those who had diagnostic records of glaucoma (ICD-10 codes, H40.1–H40.9, H42.0, H42.8, and Q15.0) within a 1-year look-back period.	HR: 1.04 (1.02–1.07)	Moderate
Ngo (24)	2013	India	115	60.86	65	Prospective	Age of 30 years or older, OAG in the study eye as determined by a glaucoma specialist, best corrected by early treatment Diabetic Retinopathy Study visual acuity of 20/60 or better in the study eye, and acceptable reliability indexes in previous visual fields (VF) performed.	IOP decreased: Normal weight (−1.5, 95%CI -2.7–(−0.4)); Overweight (−1.9, 95%CI -3.4–(−0.4)); Obese (−2.5, 95%CI -3.9–(−1.2))	Moderate
Chen (25)	2021	China	11,939	66.7	≥18	Retrospective	Individuals aged ≥18 years.	HR(95% CI): 1.54 (1.23–1.94)	Moderate
Rosa (26)	2024	African	6,634	NA	NA	Retrospective	The POAAGG study population consists of 10,255 subjects of African ancestry (Black, Afro-Caribbean, or African American) who were over the age of 35 and were recruited from greater Philadelphia. Each enrolled subject was classified by a glaucoma specialist or ophthalmologist as a POAG case, POAG suspect, or control based on previously determined criteria.	OR(95%CI) 1.02 (1.007–1.023), *p* = 0.0003	Moderate
Lee (27)	2019	Korea	287,553	NA	>40	Prospective	From the KNHIS-NSC 2002 to 2013 project, we included subjects who had undergone a general health examination at least once between 2002 and 2008 (*n* = 424,712). Subjects were identified as having POAG if they had at least two visits for POAG (International Classification of Diseases [ICD]-10 code H40.1) and received antiglaucoma medications during the study period.	HR: 1.35 (1.16–1.56)	Moderate
Lin (28)	2018	Korea	10,978	NA	>40	Cross-sectional	All participants had measured intraocular pressure of <22 mmHg and open anterior chamber angles. OAG was defined using disc and visual field criteria established by the International Society for Geographical and Epidemiological Ophthalmology.	Lower vs. normal BMI OR: 2.28 (1.22–4.26)	Moderate
Zhao (29)	2023	Korea	13,357	NA	>45	Retrospective	This study analyzed data from CHARLS, a nationally representative longitudinal survey of people in China aged 45 years or older and their spouses. In total, 150 county-level units from 28 provinces were randomly selected from a sampling framework that included all county-level units except Tibet.	HR: 0.90, 95% CI: 0.83–0.97	Moderate
Wise (30)	2011	USA	59,000	100	21–69	Prospective	The BWHS is an ongoing U. S. prospective cohort study, established in 1995 when 59,000 African-American women aged 21–69 years were enrolled through mailed questionnaires.	Age: <50 HR: 3.96 (0.94–16.7); ≥ 50 1.38 (0.83–2.30)	Moderate
Pasquale (31)	2010	USA	120,129	65.6	NA	Prospective	78,777 women in the Nurses’ Health Study and 41,352 men in the Health Professionals Follow-up Study.	HR: 0.98 (0.76–1.26)	High
Ko (32)	2016	USA	5,746	NA	≥ 40	Cross-sectional	Data from the NHANES from 2005 to 2008, are presentative sample of the population in the United States.	OR: 1.63 (1.10–2.41)	Moderate
Cohen (33)	2016	USA	18,575	32	46 ± 10	Retrospective	The subjects attending the center include men and nonpregnant women within an age range of 20 to 80 years.	Men OR: 2.86 (2.09–3.91); Women OR: 2.73 (1.62–4.60)	Moderate
Jang (34)	2014	Korea	15,271	56.8	>19	Cross-sectional	A total of 15,271 subjects (6,600 men and 8,671 women) participated from 2008–2010 in the Korea National Health and Nutrition Examination Survey.	Men OR: 1.64 (1.31–2.05); Women OR: 1.40 (1.09–1.79)	Moderate
Kyari (35)	2016	Nigeria	13,200	54.3	≥ 40	Cross-sectional	The Nigeria Blindness Survey gave a national representative sample of 15,375 persons aged 40 years and above in 310 clusters across the country.	Overweight OR: 0.82 (0.58–1.17); Obese OR: 1.18 (0.71–1.96)	Weak

BMI, body mass index; OR, odds ratio; HR, hazard ratio; CI, confidence intervals.

### Obesity and risk of glaucoma

**Figure 2:**
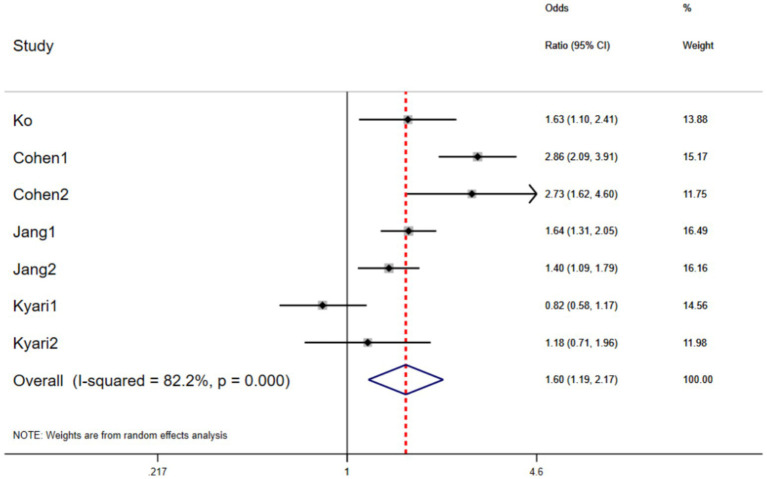
The forest plots of the odds ratio (OR) and 95% CIs for the relationship between obesity and glaucoma (*p* = 0.002).

**Figure 3:**
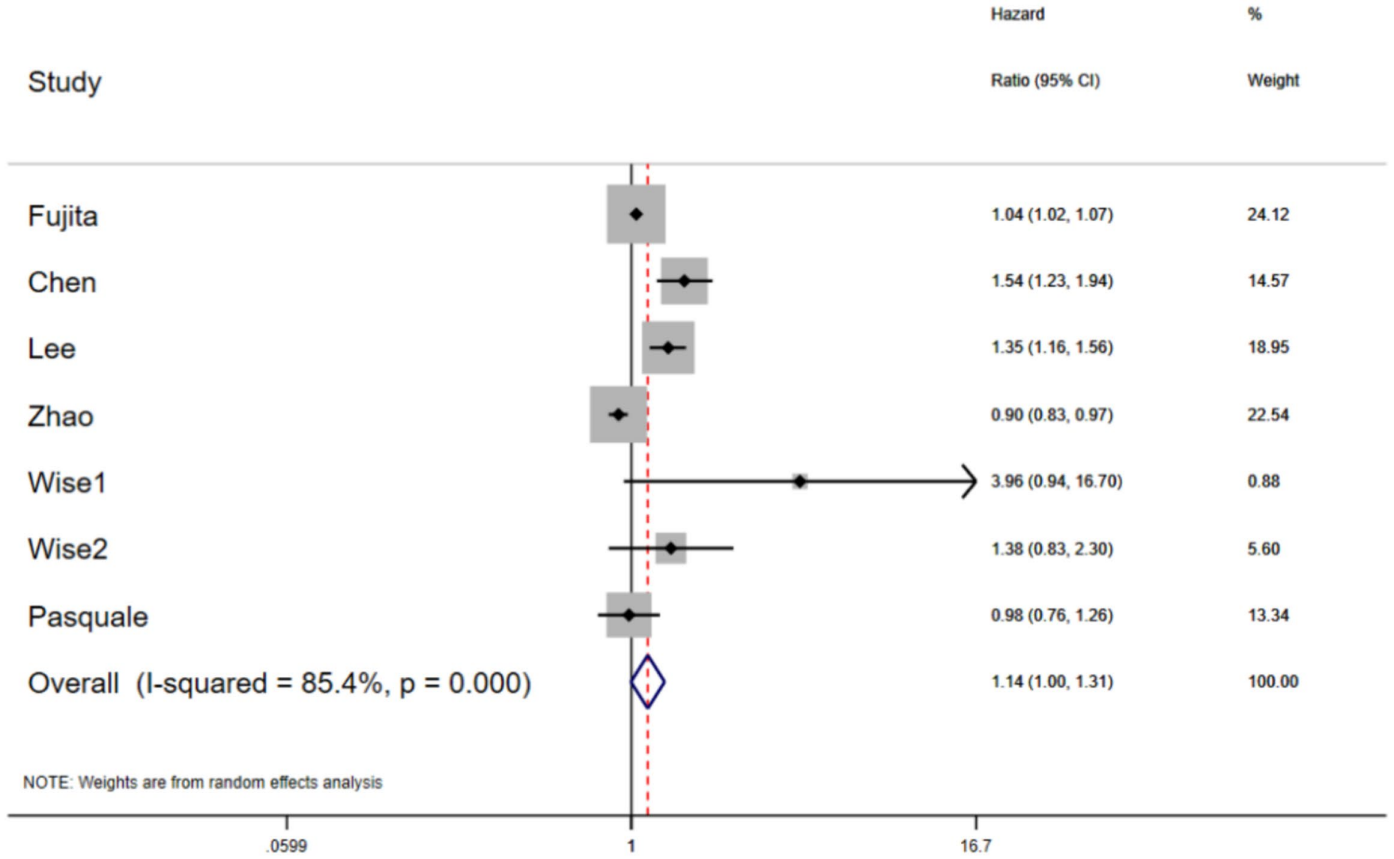
The forest plots of the hazard ratio (HR) and 95% CIs for the relationship between obesity and glaucoma (*p* = 0.05).

Pooling the existing evidence, we found that compared with individuals of normal weight, those with obesity or overweight had a 60% higher risk of glaucoma (OR: 1.60 [95% CI: 1.19–2.17], *p* = 0.002; prediction interval: 1.05–2.46), although significant heterogeneity was observed (*I*^2^ = 82.2%, *p* < 0.001) (Figure 2). In terms of the HR values, the risk of glaucoma in individuals with obesity or overweight was not significantly higher than in those of normal weight (HR: 1.14 [95% CI: 1.00–1.31], *p* = 0.058) (Figure 3). In addition, one study reported that in individuals with open-angle glaucoma of normal weight, changes in systolic blood pressure were positively correlated with changes in intraocular pressure (IOP), a relationship not observed overweight or obese individuals (Table 2).

### Subgroup analysis

To further explore sources of heterogeneity, subgroup analyses were conducted based on country, sex, and age. Compared with individuals of normal weight, those with obesity or overweight in developed countries had a 91% higher risk of glaucoma (OR: 1.91 [95% CI: 1.44–2.52], *p* < 0.001; prediction interval: 1.28–2.83), whereas in the developing countries the association was not significant (OR: 0.72 [95% CI: 0.40–1.30], *p* = 0.27) (Table 3). Individuals with obesity or overweight who are aged ≥ 40 did not show a higher risk of glaucoma than those of normal weight (OR: 1.65 [95% CI: 0.99–2.75], *p* = 0.06) (Table 3). In addition, compared with individuals of normal weight, obese or overweight men had a 114% higher risk of glaucoma (OR: 2.14 [95% CI: 1.24–3.69], *p* = 0.006; prediction interval: 0.99–4.63), while the association was not significant in women (OR: 1.88 [95% CI: 0.98–3.59], *p* = 0.06) (Table 3).

**Table 3:** The subgroup analysis of odds ratio for the relationship between obesity and glaucoma.

Subgroup	No. of studies	OR (95% CI)	*P*-value	Heterogeneity		Prediction interval
				I^2^ (%)	*P*-value	
Country						
Developed	5	1.91 (1.44–2.52)	<0.001	74.5	0.003	1.28–2.83
Developing	2	0.72 (0.40–1.30)	0.27	81.0	0.005	0.31–1.66
Age						
≥40	5	1.65 (0.99–2.75)	0.06	87.6	<0.001	0.80–3.40
Sex						
Men	2	2.14 (1.24–3.69)	0.006	87.5	0.005	0.99–4.63
Women	2	1.88 (0.98–3.59)	0.06	80.5	0.02	0.75–4.70

OR, odds ratio.

Furthermore, we also examined HR values for relationship between obesity and glaucoma. In both developed and developing countries, the HR values of glaucoma in individuals with obesity or overweight was not significantly higher than those of normal weight (developed HR: 1.07 [95% CI: 0.91–1.26], *p* = 0.43; developing HR: 1.22 [95% CI: 0.86–1.73], *p* = 0.27) (Table 4). In addition, individuals aged ≥ 40 or women with obesity or overweight did not exhibit significantly higher HR values (age ≥ 40 HR: 1.07 [95% CI: 0.94–1.21], *p* = 0.34; women HR: 1.87 [95% CI: 0.73–4.77], *p* = 0.19).

**Table 4:** The subgroup analysis of the hazard ratio for the relationship between obesity and glaucoma.

Subgroup	No. of studies	HR (95% CI)	*P* value	Heterogeneity		Prediction interval
				I^2^ (%)	*P*-value	
Country						
Developed	4	1.07 (0.91–1.26)	0.43	36.3	0.19	0.85–1.35
Developing	3	1.22 (0.86–1.73)	0.27	94.5	<0.001	0.74–2.00
Age						
≥ 40	5	1.07 (0.94–1.21)	0.34	84.8	<0.001	0.89–1.27
Sex						
Women	2	1.87 (0.73–4.77)	0.19	45.4	0.18	0.49–7.04

HR, hazard ratio.

### Publication bias

Egger’s and Begg’s tests were used to assess publication bias. The results showed no obvious publication bias (Egger: OR *p* = 0.94, HR *p* = 0.33; Begg: OR *p* = 0.88, HR *p* = 0.65) (Supplementary Figures 1–4).

### Sensitivity analysis

Sensitivity analysis of the relationship between obesity and glaucoma showed that no study significantly affected the final pooled OR HR values (Supplementary Figures 5, 6).

## Discussion

The most important contribution of this study is the finding that obesity or overweight substantially increases glaucoma risk. Subgroup analysis further suggest that the risk of glaucoma is higher among individuals with obesity or overweight in developed countries and among obese men.

Although the pathogenesis of glaucoma remains unclear, some studies have suggested that the mechanisms by which obesity increases the risk of glaucoma may include: (1) certain connection between glaucoma and high IOP, which may be related to the hypertension caused by high BMI (36, 37). Moreover, the increased red blood cell aggregation, hematocrit, and hemoglobin levels in obese individuals make their blood more viscous, thereby increases IOP by resistance to the outflow of aqueous humor from the eye (38, 39). (2) Vascular dysregulation and vasospasm can lead to reduced blood flow to the optic nerve and also cause increased IOP (40, 41). (3) The gut microbiome is considered a potential environmental factor, and obesity is often accompanied by gut microbiota dysbiosis (10). By pooling the existing evidence, this study demonstrates that obesity or overweight significantly increases glaucoma risk. Therefore, effective measures to prevent glaucoma include reasonably adjusting the diet and maintaining BMI within the normal level.

Compared with the study by Liu et al. (18), this study explores the relationship between obesity or overweight and glaucoma risk from two perspectives: OR values and prediction interval. The results indicate that obesity or overweight significantly increases glaucoma risk, consistent with some previous epidemiological findings (16, 25, 36). In addition, compared with the study by Khan et al. (42), this study provides detailed subgroup analyses based on country, age, and sex. The findings suggest that glaucoma risk is elevated among individuals with obesity or overweight in developed countries and among obese men. Furthermore, a study has shown that low BMI is also associated with increased glaucoma risk, potentially due to nutritional deficiencies and imbalances in fat-related factors (26, 28, 43).

Subgroup analysis further revealed that the risk of glaucoma in individuals with obesity or overweight in developed countries is higher than that in developing countries. This may be due to the combined effect of various interrelated pathophysiological mechanisms, such as gut microbiota dysbiosis and adipokine imbalance (10, 44). Gut microbiota dysbiosis increases intestinal permeability, leading to the accumulation of microbes and metabolites in the central nervous system, which promotes neurodegeneration and accelerates glaucoma progression (45, 46). Dysbiosis also disrupts the secondary bile acid pool, contributing to immune dysregulation, increased oxidative stress, and optic nerve damage. These mechanisms are closely related to lifestyle and dietary habits. In addition, the risk of glaucoma increased in men with obesity or overweight may be related to the differences in body composition between men and women. Estrogen in women may play an important neuroprotective role by regulating smooth muscle tone and vascular resistance to affect aqueous humor production and outflow (47). Moreover, relevant studies show that adipose tissue, as an endocrine organ, may affect retinal ganglion cell health by secreting other paracrine factors (44). Finally, the specific mechanisms by which obesity affects glaucoma require further investigation.

In addition to CI, this study also applied the prediction interval to evaluate the reliability of the results, thereby providing a more comprehensive and practical prediction outcome for related studies (21). Moreover, the heterogeneity in this study is more evident in the subgroup analysis. In the population stratified by age and sex, the I^2^ statistic fluctuates significantly. However, the sensitivity analysis did not show that any single study significantly affected the final pooled OR value. This may be related to the significant heterogeneity among populations in different regions.

Additionally, the pooled OR analysis in this study exhibited high heterogeneity. This study employed meta-regression analysis to investigate the heterogeneity. The results suggest that region (*p* = 0.015) and sex (*p* = 0.027) may be contributing factors to the high heterogeneity in the outcomes, associated with potential differences in genetic backgrounds and environmental exposures.

This study incorporated OR and HR values to evaluate the relationship between obesity or overweight and glaucoma risk. The OR value indicates that obesity or overweight is associated with a higher risk of glaucoma, while the HR value showed no significant correlation. The reasons for this discrepancy may be as follows: (1) glaucoma is a low-incidence event. Although many cohort studies included large sample sizes, the actual number of glaucoma cases was relatively small, which may have led to insufficient statistical power. This does not necessarily imply that obesity is unrelated to glaucoma risk. (2) Obesity influences glaucoma development through mechanisms such as IOP, vascular blood flow, and oxidative stress, which typically require a longer period to manifest. This may affect the outcomes of longitudinal studies, whereas cross-sectional studies might be less impacted.

This study has certain limitations. First, only cross-sectional, prospective and retrospective studies were included; well-designed and large-scale prospective cohort studies are necessary. Second, uncontrollable factors may influence the results. For example, differences in the obesity criteria or glaucoma diagnostic criteria across studies may have a certain impact on the final results. Third, high heterogeneity observed among studies, with varying definitions for obesity and glaucoma. Finally, despite assessment, the presence of publication bias remains incomplete.

## Conclusion

We found that obesity or overweight increases glaucoma risk in term of OR values. Individuals with obesity or overweight in developed countries, as well as obese men, appear to be associated with higher risk. Therefore, maintaining weight within the normal range may be an effective measure to prevent glaucoma.

## Data Availability

The original contributions presented in the study are included in the article/Supplementary material, further inquiries can be directed to the corresponding authors.
