# Supplementary material for: The impact of obesity or overweight on the risk of glaucoma: a meta-analysis
Source: Front Med (Lausanne). 2026 Jan 30;13:1756819. doi: 10.3389/fmed.2026.1756819 (PMC12900665; doi:10.3389/fmed.2026.1756819)
Supplement: Supplementary file 1 [file Supplementary_file_1.docx]

**Figure 1** The Begg's test for publication bias of the odds ratio. p = 0.88

**Figure 2** The Begg's test for publication bias of the hazard ratio. p = 0.65


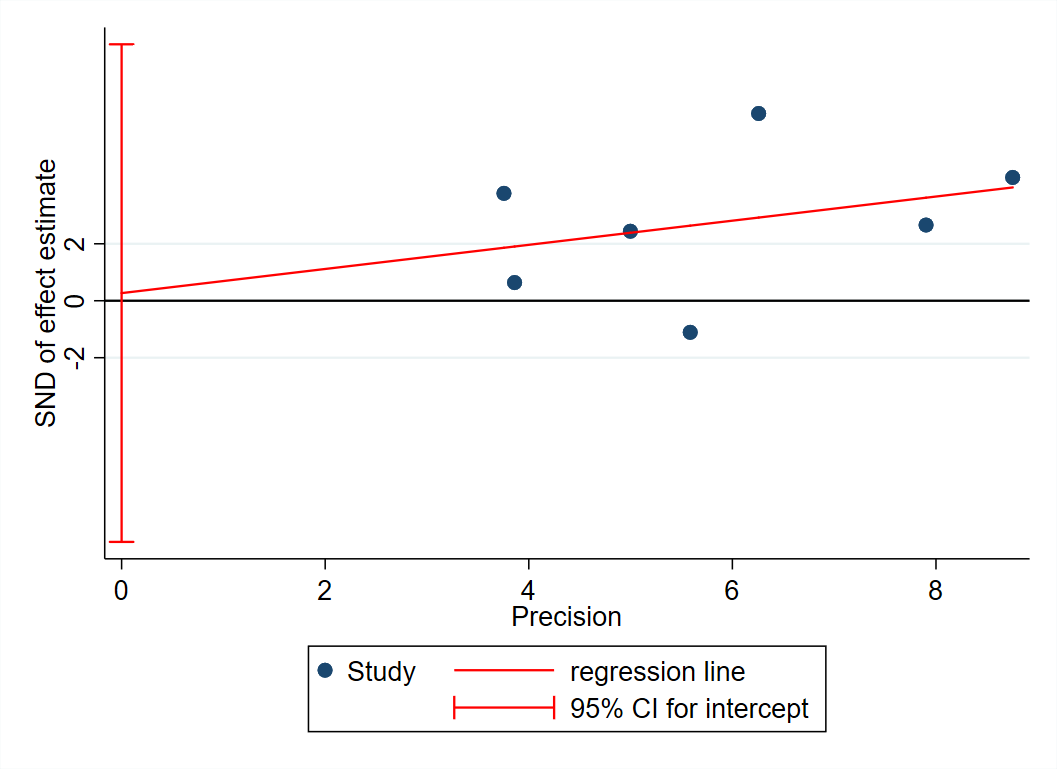


**Figure 3** The Egger's test for publication bias of the odds ratio. p = 0.94


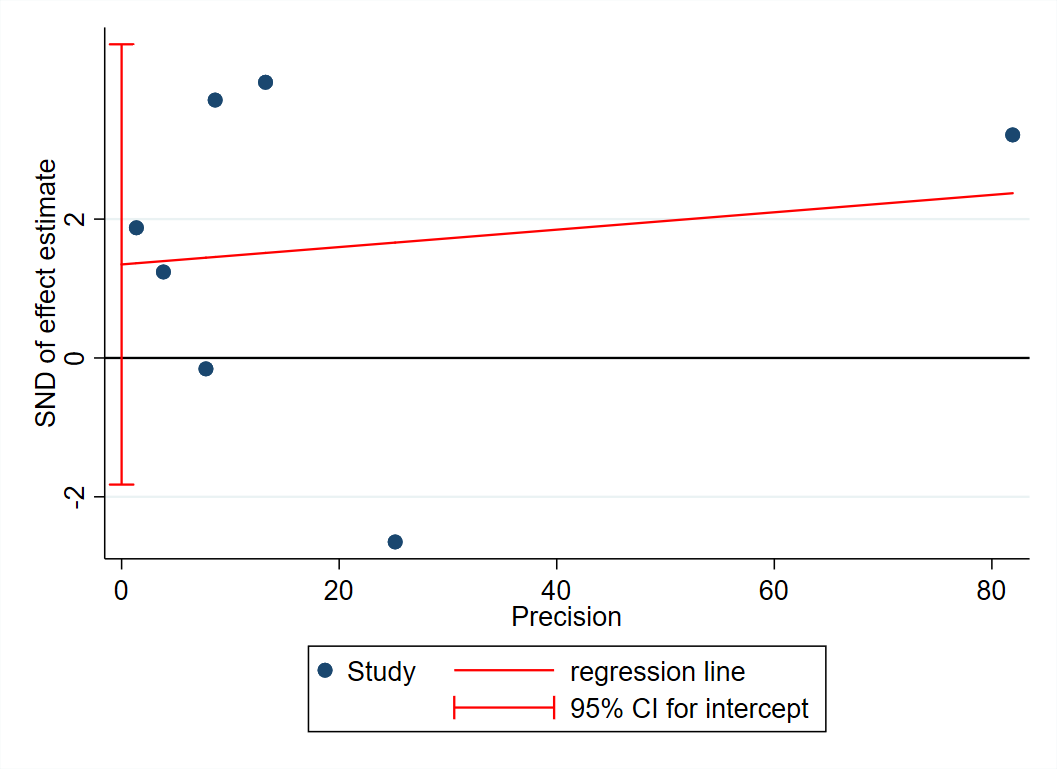


**Figure 4** The Egger's test for publication bias of the hazard ratio. p = 0.33

**Figure 5** The sensitivity analysis of the odds ratio.

**Figure 6** The sensitivity analysis of the hazard ratio.
